# Genome-wide analysis of long non-coding RNAs affecting roots development at an early stage in the rice response to cadmium stress

**DOI:** 10.1186/s12864-018-4807-6

**Published:** 2018-06-15

**Authors:** Liang Chen, Shilai Shi, Ninfei Jiang, Hira Khanzada, Ghulam Mustafa Wassan, Changlan Zhu, Xiaosong Peng, Jie Xu, Yujin Chen, Qiuying Yu, Xiaopeng He, Junru Fu, Xiaorong Chen, Lifang Hu, Linjuan Ouyang, Xiaotang Sun, Haohua He, Jianmin Bian

**Affiliations:** 1 Key Laboratory of Crop Physiology, Ecology and Genetic Breeding, Ministry of Education. Jiangxi Agricultural University, Nanchang, 330045 China; 2 Southern Regional Collaborative Innovation Center for Grain and Oil Crops in China, Nanchang, China; 3 College of Agronomy, Jiangxi Agricultural University, Nanchang, 330045 China

**Keywords:** Rice, Cd stress, lncRNA, mRNA, Cis, Trans

## Abstract

**Background:**

Long non-coding RNAs (lncRNAs) have been found to play a vital role in several gene regulatory networks involved in the various biological processes in plants related to stress response. However, systematic analyses of lncRNAs expressed in rice Cadmium (Cd) stress are seldom studied. Thus, we presented the characterization and expression of lncRNAs in rice root development at an early stage in response to Cd stress.

**Results:**

The lncRNA deep sequencing revealed differentially expressed lncRNAs among Cd stress and normal condition. In the Cd stress group, 69 lncRNAs were up-regulated and 75 lncRNAs were down-regulated. Furthermore, 386 matched lncRNA-mRNA pairs were detected for 120 differentially expressed lncRNAs and 362 differentially expressed genes in cis, and target gene-related pathway analyses exhibited significant variations in cysteine and methionine metabolism pathway-related genes. For the genes in trans, overall, 28,276 interaction relationships for 144 lncRNAs and differentially expressed protein-coding genes were detected. The pathway analyses found that secondary metabolites, such as phenylpropanoids and phenylalanine, and photosynthesis pathway-related genes were significantly altered by Cd stress. All of these results indicate that lncRNAs may regulate genes of cysteine-rich peptide metabolism in cis, as well as secondary metabolites and photosynthesis in trans, to activate various physiological and biochemical reactions to respond to excessive Cd.

**Conclusion:**

The present study could provide a valuable resource for lncRNA studies in response to Cd treatment in rice. It also expands our knowledge about lncRNA biological function and contributes to the annotation of the rice genome.

**Electronic supplementary material:**

The online version of this article (10.1186/s12864-018-4807-6) contains supplementary material, which is available to authorized users.

## Background

Long noncoding RNAs (lncRNAs) are a diverse class of molecules derived from RNA polymerases and arise as important regulators in the regulation of gene expression and transcription in animals and plants [1]. LncRNAs are also involved in the regulation of targeted genes via epigenetic, transcriptional and post-transcriptional methods [2]. LncRNAs are clearly distinguishable from mRNAs in their sequence structure, expression level, positional characteristics and evolution conservation [3–5]. Furthermore, their subspecies have been characterized and categorized in humans [3, 6], *Caenorhabditis elegans* [7] and zebrafish [8]. In recent years, studies on lncRNAs representing different classes of transcripts longer than 200 nucleotides have extended our understanding of the eukaryotic transcriptome [9]. LncRNAs can help recruit the PHD-PRC2 complex to enable histone modifications of FLC via epigenetic regulation [10]. Nuclear speckle RNA-binding proteins (NSRs), together with AS competitor long noncoding RNAs, also called ASCO-lncRNAs, can interfere with AtNSRs in alternative splicing of auxin-responsive genes downstream and affect the growth of lateral roots [11]. In plants, numerous molecular functions and biological processes have been determined by lncRNAs; for example, vernalization, photomorphogenesis, fertility, protein re-localization, alternative splicing, phosphate homeostasis, modulation of chromatin loop dynamics etc. [12] Over the last decade, the role and function of small non-coding RNAs in plants have been widely studied [13]. However, the functional mechanisms of lncRNAs in several plant species remain unexplored and only a few of lncRNAs have been fully characterized until now. In Arabidopsis, lncRNAs such as cold-assisted intronic noncoding RNA (COLDAIR) and cold induced long antisense intragenic RNA (COOLAIR) have been confirmed to facilitate chromatin altering activities in transcriptional silencing of FLC during vernalization [10, 14]. LncRNAs have been recognized to play an important role in many gene regulatory networks tangled in several biological processes of plant stress responses [2]. Moreover, a large number of putative stress lncRNAs have been categorized and characterized in Arabidopsis [15], maize [16], wheat [17], *Populus trichocarpa* [18], cucumber [19], tomato [20], cotton [21] and other plant species [22–24]. Recently, thousands of lncRNAs associated with rice development have been identified [25, 26], and could provide a guidance that rice lncRNAs function cannot be ignored. However, the elusive role of lncRNAs in rice stress responses is still not fully described or understood. Therefore, it is necessary to investigate the function of lncRNAs in rice stress responses.

Cadmium (Cd) is known to be an unnecessary metal element for plants and extensively spread Cd pollution has significantly affected human health in terms of its direct effects on crop production and its high increase in the edible part of crops such as rice [27]. When lncRNA function is well understood, scientists may realize that Cd-regulated lncRNAs may be involved in heavy metal stress responses, and some Cd-regulated lncRNAs have recently been detected [28]. Although previous studies have provided useful information on the mechanisms for rice lncRNA in Cd stress response, the regulatory mechanisms involved are mostly unknown. To improve our understanding of the likely functional roles of lncRNAs in rice Cd stress response, further studies are necessary to understand the functional genetics of lncRNAs in detail, and to determine which specific lncRNAs target selective sites for interaction in the rice genome.

Inclusive identification of plant lncRNAs at the genomic level is mainly dependent on and determined by advances in technical platforms [2]. Genome-wide screening by high-throughput RNA sequencing and computational applications for inclusive identification of lncRNAs would be a better choice for detailed mapping and structural studies to understand the RNA-protein and RNA-DNA interactions [29]. In plants, genome-wide analysis of lncRNAs with RNA sequencing transcriptomic data has been executed in only a limited plant species such as *Arabidopsis thaliana* [30–32], *Triticum aestivum* [22], *Oryza sativa* [25], tomato [33] and *Zea mays* [16, 34].

In the current study, we used a deep RNA sequencing strategy to clarify the lncRNAs profiled associated with Cd stress using the Cd response rice genotype which could provide more insights into the regulatory role of more lncRNAs in the rice Cd stress response. The results obtained in our study provided a valuable resource to study the lncRNAs involved in Cd stress response and will increase the knowledge for a better understanding of the biological processes of rice stress response.

## Methods

### Plant material and growth conditions

One rice genotype (DX142) was selected from 82 different screened rice genotypes to measure phenotypic traits and for gene expression in this study. DX142 is a pure line and shows the highest sensitivity to Cd stress.

Full seeds of DX142 were surface sterilized with 0.5% NaClO solution for 30 min, rinsed five times with distilled water and maintained for 2 days at 25–30 °C in the dark, thereby inducing germination. Seedlings were then exposed to treatments without 100 mg/L CdCl_2_(as a control) and with 100 mg/L CdCl_2_. Growth conditions were as follows: 16/8 h day/night photoperiod under 28/25 °C day/night temperatures. Roots were collected for gene expression analysis on the 5th day after treatments.

### RNA extraction, sequencing and database access

Total RNA was extracted from 5-day-old rice roots with Trizol reagent (Invitrogen, Carlsbad, CA, USA). Six samples (three replicated samples for each treatment; CK_R1, CK_R2 and CK_R3 for the normal condition: SY_R1, SY_R2 and SY_R3 for the stress condition) were used for sequencing, and differentially expressed lncRNAs and mRNAs were obtained.

RNA isolation, quantification and library preparation for lncRNA sequencing, clustering and sequencing and quality control were analysed as described by Ren et al. (2016) [35]. After these analyses, the purified data with high quality were mapped to the reference genome of *Japonica* variety Nipponbare [36] using Bowtie v2.0.6 and TopHat v2.0.9 software [37]. The clean data were uploaded into the NCBI Sequence Read Archive under the accession number SRP099996. The mapped reads of each sample were assembled by both Scripture (beta2) [38] and Cufflinks (v2.1.1) [39] in a reference-based approach. Cuffdiff (v2.1.1) was used to calculate FPKMs (fragments per kb per million reads) of lncRNAs in each sample [39]. Furthermore, the negative binomial distribution method was used to detect the lncRNAs obtained from the three biological replicates of each treatment. Finally, the lncRNAs developed were referred to as Cd stress (SY_R) and control (CK_R)-related lncRNAs.

### lncRNA identification pipeline

To attain putative lncRNAs, we initially filtered the transcripts according to the class code annotated by Cuffcompare; only the transcripts that occurred in at least two samples were retained. To acquire lncRNAs, we only retained novel (not overlapping with known genes in sense), large (longer than 200 nucleotides), expressed (for multiple-exon transcripts FPKM ≥0.5, for single-exon transcripts FPKM ≥2) transcripts. Then, to obtain high-quality data, CPC (0.9-r2) [40] and Pfam-scan [41] were used to identify the candidate lncRNAs. Transcripts with coding potential predicted by any of the two tools previously described were filtered out, and those without coding potential were retained. Finally, we selected those shared by the two tools as the final candidate lncRNAs and used them for further analysis.

### Identification of differentially expressed lncRNAs

The fragment per kb per million reads (FPKM) for lncRNAs was calculated by using Cuffdiff (v2.1.1) software [42]. For biological replicates, transcripts or genes with an adjusted *p*<0.05 (*q*<0.05) were designated differentially expressed among the two groups of rice roots.

### LncRNAs target gene prediction and functional enrichment analysis

We searched coding genes 10 kb/100 kb upstream and downstream of lncRNAs as the cis target gene, and then analysed their function. The trans role of lncRNAs was identified by the expression level. We calculated the expressed correlation between lncRNAs and coding genes with custom scripts; further, we clustered the genes from different samples with WGCNA [43] to search for common expression modules and then analyzed their function through functional enrichment analysis.

To understand the functional roles of the target genes of lncRNAs, we used the Gene Ontology (GO) seq [44] R package to implement enrichment analysis, in which gene length bias was corrected. In addition, Kyoto Encyclopedia of Genes and Genomes (KEGG) enrichment analysis on target genes was performed on KOBAS software [45] using a hypergeometric test. GO terms and KEGG pathways with a corrected *p*<0.05 (q<0.05) were considered significantly enriched.

### Real-time quantitative PCR

Total RNA from 6 sequenced samples (include 3 biological duplication 2 treatment) were extracted using a PrimeScript™ RT reagent Kit with gDNA Eraser. SYBR-based qRT-PCR reactions (SYBR Green I, Osaka, Japan) were performed on a ABI VIIA@7 using the following reaction conditions: 95 °C for 10 min followed by 40 cycles at 95 °C for 15 s and 60 °C for 30s. All qRT-PCR reactions were performed in triplicate samples, and the results were analyzed with the system’s relative quantification software (ver.1.5) based on the (ΔΔCT) method. The detection of threshold cycle for each reaction was normalized against the expression level of the rice ACTB gene.

## Results

### Morphology under cd stress and control conditions

First, 82 different rice genotypes were used in this study to check their Cd stress sensitivity for acquiring additional insights into the rice transcriptomic response to environmental Cd stress. One genotype DX142, showed the highest sensitivity and was used for further analyses.

**Fig. 1:**
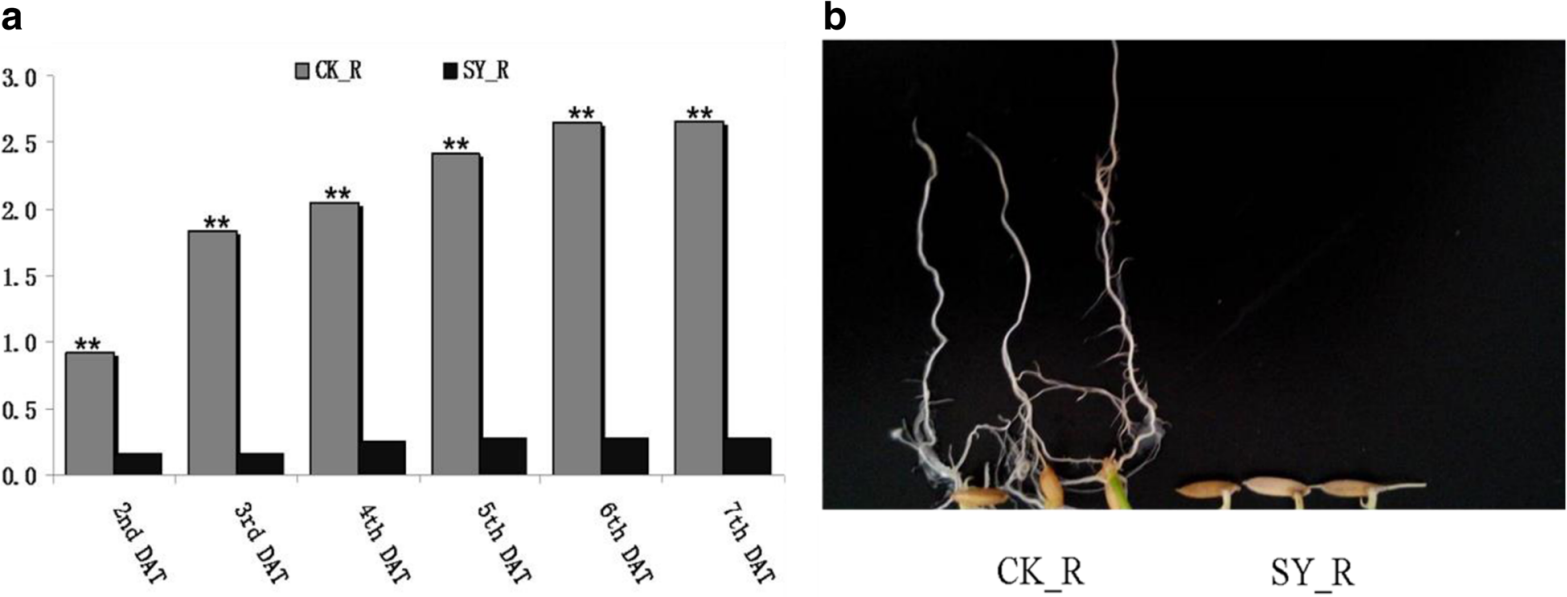
Root length of DX142 under different treatments. **a** The root length under Cd treatment compared with control. ** mean the significant levels of 1%, DAT means days after treatment; **b** The root appearance of DX142 under Cd treatments and control condition at 5th DAT

The average root length of DX142 under Cd stress and control conditions is significantly different. The average root length of DX142 under the control condition was significantly higher than under the Cd stress condition from the second day (Fig. 1a), indicating that the rice root length was obviously inhibited by Cd. Furthermore, the root length of DX142 under control condition increased slowly after six days. Therefore, to obtain as much as lncRNA information as possible regarding rice Cd stress, the roots in five-day-old roots were harvested to detect lncRNAs and for further analysis (Fig. 1b).

### Identification of lncRNAs

A total of 679,838,650 raw reads were generated using the Illumina HiSeq 2000 platform. Low-quality paired-end sequences and adapter sequences were trimmed off, and 660 million clean reads (99 Gb) were obtained with an average of 110 million reads (16.5 Gb) per sample (Table 1). Subsequently, we mapped the clean reads to the Nipponbare reference genome [36] to identify the transcripts.

**Table 1:** Statistics of the read alignments in the RNA-Seq study

Sample name	Raw reads	Clean reads	clean bases	Error rate (%)	Q20(%)	Q30(%)	GC content(%)
CK_R1	102,609,352	100,129,870	15.02G	0.01	97.57	93.83	52.22
CK_R2	102,837,492	100,135,724	15.02G	0.01	97.59	93.82	52.55
CK_R3	119,750,698	116,752,212	17.51G	0.01	97.66	93.98	52.15
SY_R1	108,004,232	104,861,548	15.73G	0.01	97.56	93.79	52.69
SY_R2	138,500,332	133,956,886	20.09G	0.01	97.65	93.97	52.72
SY_R3	108,136,544	104,207,142	15.63G	0.01	97.62	93.89	53.65

**Fig. 2:**
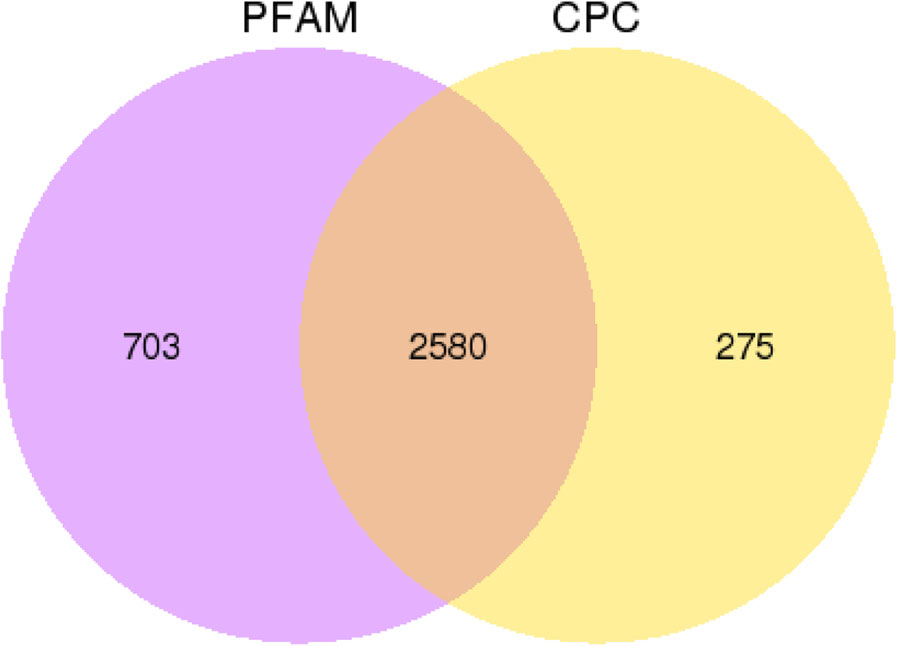
Screening of the candidate lncRNAs. Venn diagrams of coding potential analysis by using stringent criteria. Two tools (CPC and PFAM) were employed to analyse the coding potential of lncRNAs. Those simultaneously shared by two analytical tools were designated as candidate lncRNAs and used in subsequent analyses

Considering the characteristics of lncRNA sequences (≥200 nt) and their differences from other classes of RNA (mRNA, tRNA, rRNA, snRNA, snoRNA, pre-miRNA, and pseudogenes), we used Scripture (beta2) and Cufflinks (v.1.1) software to classify transcripts into different subtypes. Overall, 3558 transcripts out of all the 46,933 identified transcripts were predicted to be lncRNAs. We performed coding potential analysis using the software CPC and Pfam-scan to confirm these 3558 lncRNAs. After screening using harsh criteria and two analytic tools, a total of 2580 lncRNAs from Cd stress and control conditions in rice were identified and subjected for further analysis (Fig. 2).

### Identification of differentially expressed lncRNAs

**Fig. 3:**
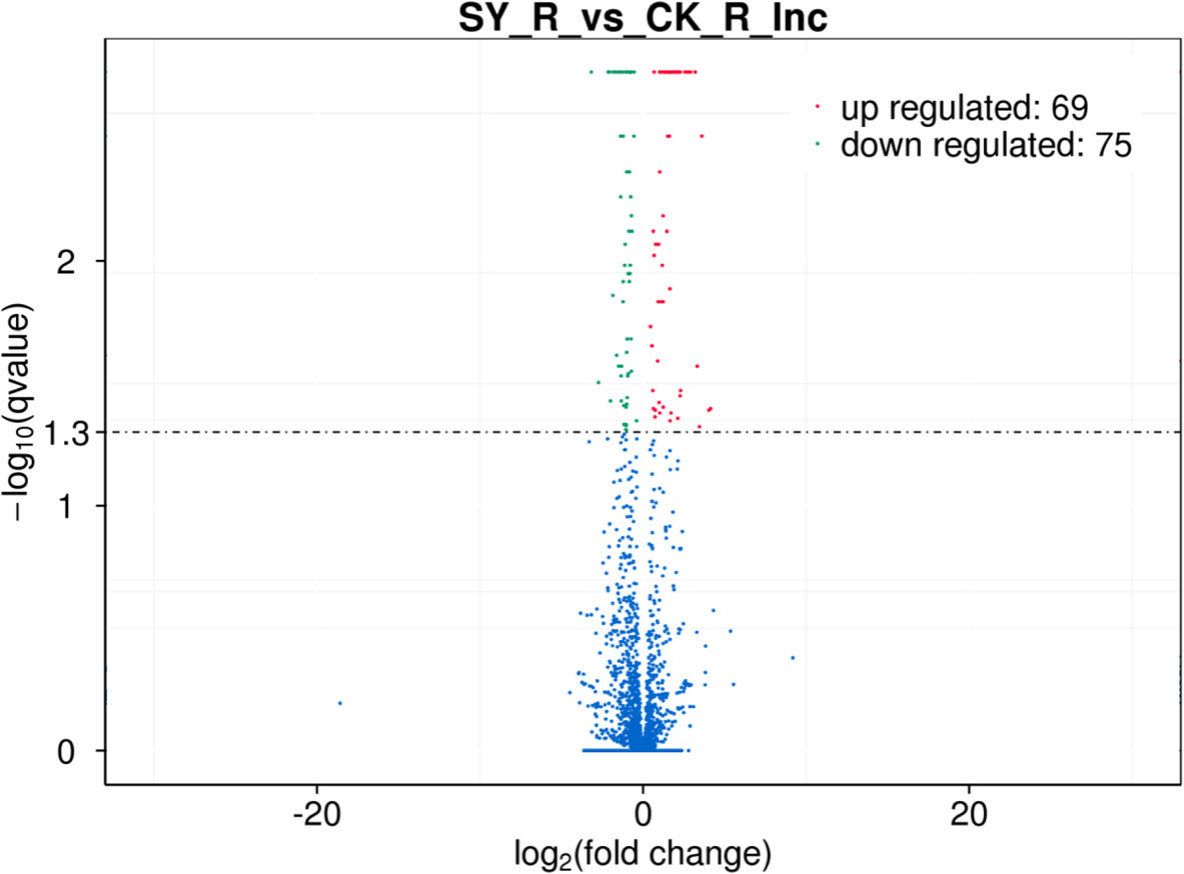
Volcano plot of significant up-and down-regulated transcription factors under Cd treatment compared with control in rice roots

Genome-wide analysis of lncRNA expression under Cd stress and control conditions was performed to profile differentially expressed lncRNAs associated with Cd stress. We first assessed the lncRNA expression profiles in two different conditions (Cd stress vs control condition), and 144 differentially expressed lncRNAs from 143 lncRNA genes were identified between Cd stress (SY_R) and control (CK_R) (Additional file 1: Table S1). The 144 lncRNAs consisted of 120 large intergenic non-coding RNAs (lincRNAs), 1 intronic lncRNA, and 23 anti-sense_lncRNAs (Additional file 1: Table S1). Among them, 69 lncRNAs were up-regulated and 75 lncRNAs were down-regulated (Fig. 3 and Additional file 1: Table S1).

### The cis role of differentially expressed lncRNAs in target genes

To examine the lncRNA function, we predicted the potential targets of lncRNAs in cis. We examined protein-coding genes 10 and 100 kb upstream and downstream of the lncRNAs, respectively. In total 386 matched lncRNA-mRNA pairs for 120 differentially expressed lncRNA genes and 362 differentially expressed mRNAs were found (Additional file 2: Table S2).

**Fig. 4:**
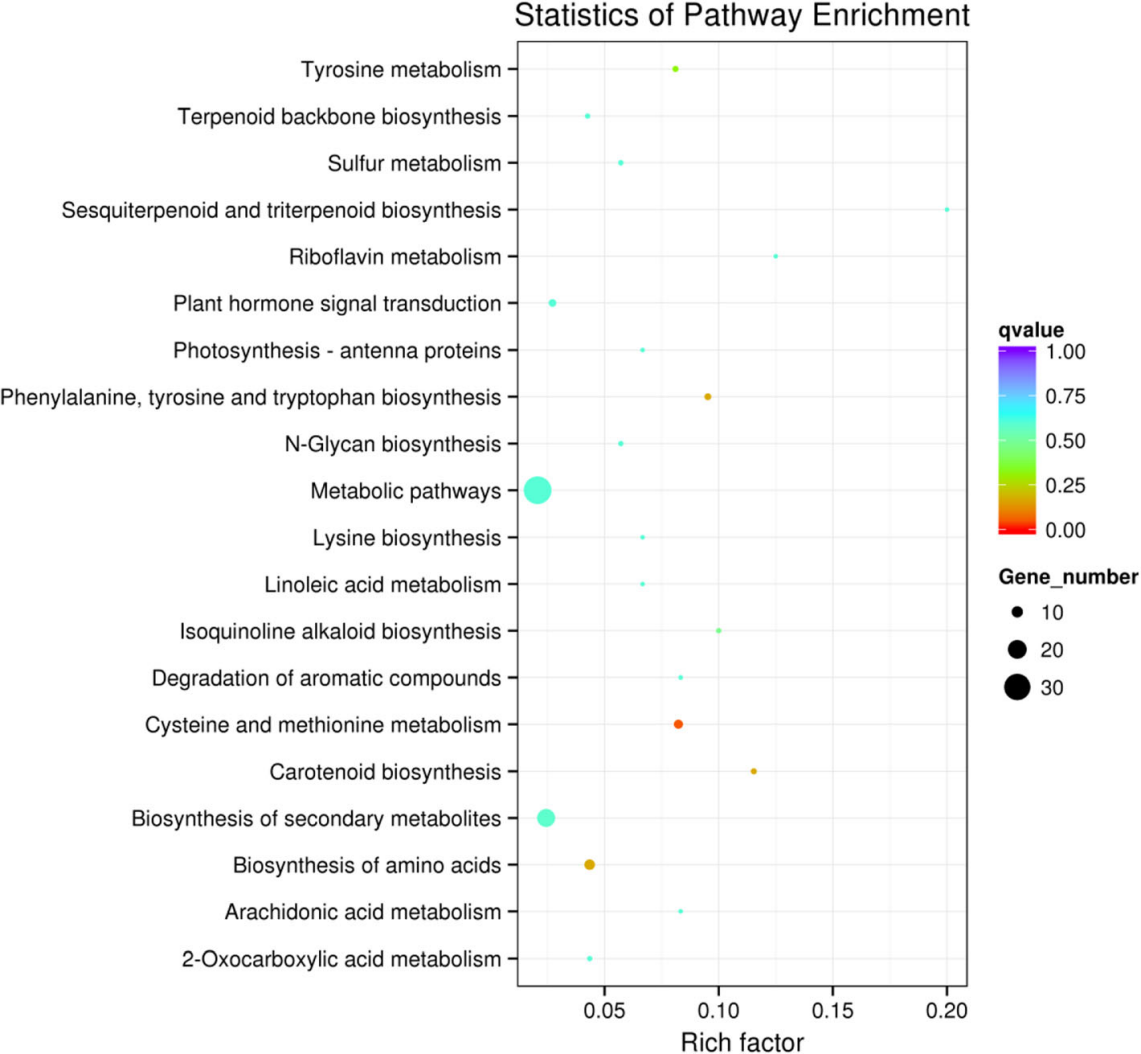
Scatter plot of KEGG pathway enrichment statistics for differentially expressed target genes in cis in rice roots. Rich Factor is the ratio of differentially expressed gene numbers annotated in this pathway term to all gene numbers annotated in this pathway term. Greater Rich Factor means greater intensiveness. *q*-value is corrected *p*-value ranging from 0~ 1, and its less value means greater intensiveness. We displayed KEGG pathways significantly enriched due to exposure to Cd in our experiments

GO analysis predicted that there was no significant enrichment in GO terms targeted by lncRNAs. The pathway analyses revealed 17 different pathways corresponding to the target genes (Table 2 and Additional file 3: Table S6); one of them is the cysteine and methionine metabolism pathway, which was significant in the Cd stress condition compared with the normal condition with an enrichment (*q* < 0.05, Fig. 4; Table 2).

**Table 2:** Significant pathways and proportions after KEGG (Kyoto Encyclopedia of Genes and Genomes) analysis of differentially expressed target genes in cis in the root. We displayed KEGG pathways significantly enriched due to exposure to Cd in our experiments

Pathway term	*q*-value	Gene number
Cysteine and methionine metabolism	0.047189	7

### The trans role of lncRNAs in target genes

On the other hand, we examined the trans role of 143 lncRNAs genes on the basis of its expressed correlation coefficient (Pearson correlation ≥0.95 or ≤ − 0.95). In a total, 28,276 interaction relationships were identified in trans between 144 lncRNAs and the protein-coding genes (Additional file 4: Table S3 and Additional file 5: Table S7).

**Fig. 5:**
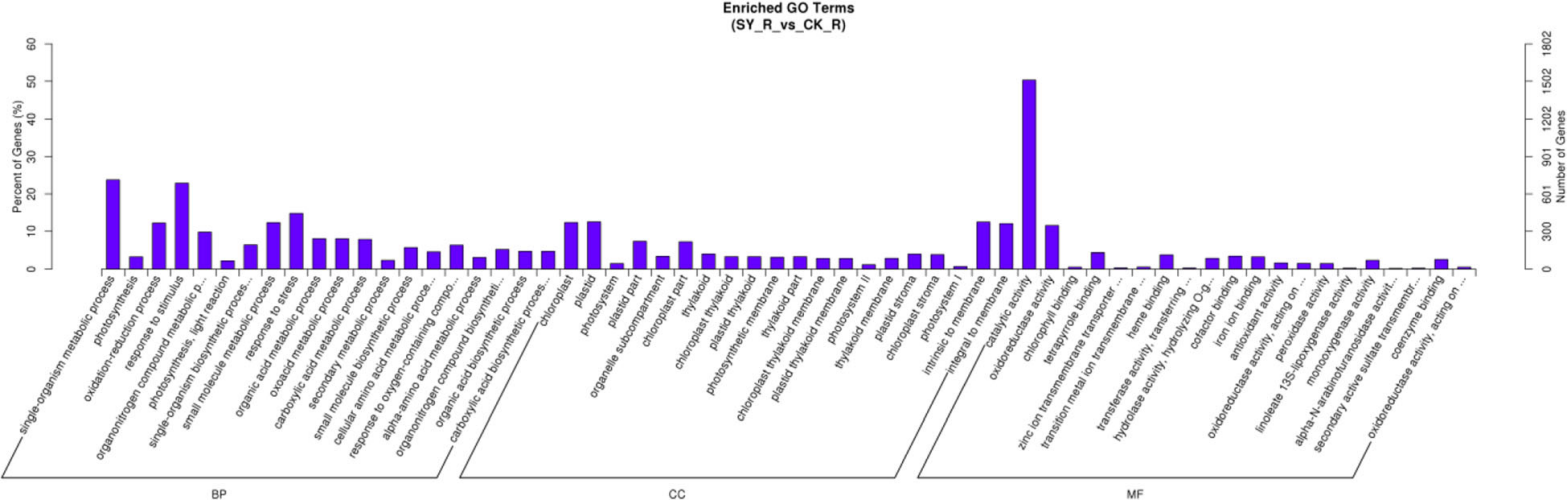
Gene Ontology (GO) enrichment analysis for differentially expressed target genes in trans in rice roots. Only the top false discovery rate ranked 20 enrichment of GO terms from “biological process, molecular function, and cellular component” categories were listed. The y-axis and x-axis indicate the number of genes in a category and the names of the clusters, respectively

GO analysis showed that the highly enriched GO terms targeted by lncRNAs are single-organism metabolic process, photosynthesis and response to stimulus (Fig. 5). The pathway analyses revealed 118 different pathways corresponding to the target genes, and four of them are biosynthesis of secondary metabolites, phenylpropanoid biosynthesis, phenylalanine metabolism and photosynthesis signalling pathways in the Cd stress condition compared with the normal condition (*q* < 0.05) (Additional file 6: Figure S1 and Table 3).

**Table 3:** Significant pathways and proportions after KEGG (Kyoto Encyclopedia of Genes and Genomes) analysis of differentially expressed target genes in trans in the root. We displayed KEGG pathways significantly enriched due to exposure to Cd in our experiments

Pathway term	*q*-value	Gene number
Biosynthesis of secondary metabolites	0.00098479	231
Phenylpropanoid biosynthesis	0.00176395	54
Phenylalanine metabolism	0.02025548	41
Photosynthesis	0.02612353	34
Photosynthesis - antenna proteins	0.0715502	11
Phenylalanine, tyrosine and tryptophan biosynthesis	0.07830629	20
Glutathione metabolism	0.07830629	31
Valine, leucine and isoleucine biosynthesis	0.07830629	10

### Validation of gene expression by quantitative real-time PCR

**Fig. 6:**
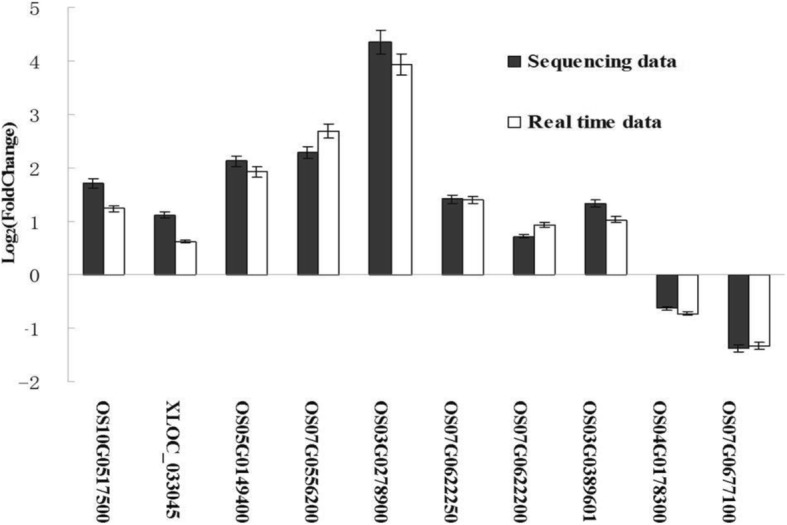
Validation of sequencing data by qRT-PCR. The sequencing data were obtained by LncRNA-seq. Because all mRNA data and lncRNA data were sequencing together and shared one mRNAs library, therefore the sequencing data of Fig. 7 were all from the data of lncRNA-seq. We made a random selection of 10 genes to determine by quantitative real-time polymerase chain reaction. The y-axis and x-axis indicate Log_2_(Fold Change) and the name of the genes, respectively. XLOC_033045 represent one of the whole lncRNAs. The Fold Change stands for the fold change between FPKM of SY and the FPKM of CK

To validate the findings from sequencing data, 10 genes correlated with mRNAs, including lncRNAs were selected randomly and analyzed by quantitative real-time polymerase chain reaction (qRT-PCR) (Fig. 6). We randomly selected 10 genes in the lncRNA-seq data. According to the random criteria, we have selected genes that were significantly different and genes that were not significantly different to enhance the reliability of the results by lncRNA-seq. The primer sequences are listed in Additional file 7: Table S4. The results showed that the expression trends were consistent for all transcripts in both analyses, with a correlation coefficient of R^2^ of 0.9643 (Additional file 8: Figure S2).

## Discussion

Although various examples of recently characterized lncRNAs [46–49] support a functional relationship between lncRNAs and their related-protein coding gene(s). The study of differentially expressed lncRNAs controlling gene expression in the rice stress response at the whole transcriptome level especially in Cd stress, is reported to be limited. Thus, our data systemically predict the lncRNAs at the whole transcriptome level and showed that which specific lncRNAs seek out selective sites in the genome for interaction in rice Cd stress. Compared to the previous studies, which were based on two or three replicated samples for each treatment for RNA sequencing, our sequencing data were obtained from three individually replicated samples for each treatment. Then, the negative binomial distribution method was used to detect the RNA information for each treatment, which could minimize the experimental error. Thus, our results could provide more comprehensive information.

Here, 143 lncRNA genes were detected that showed differential expression in the Cd stress response in the root, and 83.9 and 100% of them were identified that contained at least one differentially expressed mRNA in cis and trans, respectively, which suggests that these lncRNAs may play an important role in the rice Cd stress response. Furthermore, lncRNAs showed a higher ratio than those of the other two subtypes of all the differentially expressed lncRNAs, indicating that lncRNAs may be the main form of lncRNAs in the Cd stress response in rice. Previous studies confirmed that altered splicing of lncRNA genes could quantitatively modulate, gene expression in development and other physiological processes through co-transcriptional coupling mechanisms [50–52], but there is less information available on the effects of heavy metal stress on altered splicing regulation particularly when global lncRNA profiles are induced by treatment with certain heavy metals. In this study, 144 lncRNA transcripts from 143 lncRNA genes were identified, only one lncRNA coding gene (XLOC_057621) was alternatively spliced and yielded two different lncRNAs transcripts, and therefore it can be predicted that alternative splicing might not be the major form of regulation in the Cd stress process for rice. In addition, we compared the lncRNA data with the study of Fei et al. [28] and found that XLOC_003991 and XLOC_044902 shared the same changed pattern (Additional file 1: Table S1).

The role of lncRNAs in stress processes creates an insistence to understand the mechanisms by which these RNAs seek their targets [15–20, 22–24]. To improve the accuracy of target prediction, a detailed co-expression network between differentially expressed mRNA and differentially expressed lncRNAs was constructed, which showed that one lncRNA could target one or more coding genes. The result indicated that the regulation of mRNA by lncRNAs is involved in Cd-induced root development. Therefore, lncRNAs should be given more attention in heavy metal stress response studies in the future.

To gain more insight into the function of targets of lncRNAs, GO term and KEGG pathway annotations were applied to their target gene pool. KEGG analysis showed a significant change in the cysteine and methionine metabolism pathway in the Cd stress compared to normal condition. Plants produce cysteine-rich (Cys-rich) peptides that chelate Cd to procedure non-toxic complexes which are then sequestered into the vacuole to avoid high levels of free cytotoxic Cd in the cytosol in response to Cd stress [53]. In this study, we observed that OS03G0196600, which is involved in the cysteine and methionine metabolism pathways, was clearly up-regulated (Additional file 9: Table S5) and might contribute to the production of cysteine-rich (Cys-rich) peptides. It is interesting that XLOC_086307 (the lncRNA targeted OS03G0196600 in cis) was also up-regulated significantly, which suggests that XLOC_086307 likely participates in Cd response processes in rice by regulating the cysteine-rich peptide metabolism-related gene OS03G0196600. Previous studies showed that exposure to Cd stress will lead to impairment of the photosynthetic function in many plant species. Both chlorophylls and carotenoid contents decrease when exposed to Cd [54–56]. It was noticed that OS03G0184000 (the target of XLOC_086119 and XLOC_066284 in cis), which is involved in carotenoid biosynthesis, is up-regulated (Additional file 9: Table S5), which may result in increased carotene content. Oxidative cleavage of carotenoids will produce apocarotenoids, while the phytohormone ABA is an apocarotenoid derivative [57]. With the increase in apocarotenoids, ABA content was increased, and the signalling pathway was activated. Furthermore, ABA was found to be involved in the regulation of antioxidative defence systems and Cd-induced oxidative stress in mung bean seedlings [58]. Therefore, XLOC_086119 and XLOC_066284 might be involved in carotenoid biosynthesis associated with cadmium stress in rice.

In trans, GO and pathway analyses predicted that the regulated transcripts of lncRNAs are mainly associated with metabolic process (ontology: biological process), intracellular part (ontology: cellular component) and catalytic activity (ontology: molecular function), which are associated with four significant gene pathways that correspond to the transcripts. Among these pathways, we found that secondary metabolites such as phenylpropanoids and phenylalanine-related genes were significantly altered by Cd stress, and our results were consistent with a previous study [53]. In the current study, the trans role of lncRNAs, including XLOC_058523, XLOC_104363 and XLOC_059778, targeted phenylpropanoids and the phenylalanine related-gene OS11G0552000, which indicated that lncRNAs may regulate the genes of the secondary metabolites in far distance and then activate the various transporters to successively guide removal of excessive Cd from the cell. Furthermore, our analysis revealed that differentially expressed mRNA OS07G0148900 (Additional file 9: Table S5) with the trans targets of differentially expressed lncRNAs in trans such as XLOC_122123, XLOC_125848 and XLOC_098316, is highly enriched in photosynthesis. The results are consistent with the fact that Cd can damage the photosynthetic apparatus and lead to impairment of photosynthetic function [59, 60]. Because our samples were grown in solution, the roots may have been passively exposed to light, which could strongly activate photosynthesis in root tissues. A similar result can be seen in the study of Zhai et al. [61]. These results suggest that the photosynthesis pathway-related genes are involved in the Cd stress response in rice and may also be regulated by lncRNAs in trans. In addition, the further experiments of the interaction among these lncRNAs and their targets both in cis and trans are underway.

## Conclusions

Takn all together, our study systematically determines the genome-wide lncRNA expression profile in Cd-induced rice roots by deep sequencing. Our results showed that some lncRNAs are aberrantly expressed in Cd-treated rice roots when compared with untreated roots. In addition, after pathway analyses of the target genes of these differentially expressed lncRNAs, cysteine and methionine metabolism pathway, carotenoid biosynthesis, ABA signalling pathway (in cis), and secondary metabolites and photosynthesis (in trans) were enriched, which indicated that lncRNAs may play an important role in these pathways in response to Cd stress. Therefore, further studies are necessary needed to fully understand these lncRNAs to effectively control rice Cd pollution in the future.

## Additional files

Additional file 1: **Table S1.** Differentially expressed lncRNAs in response to Cd stress in root libraries. The log_2_(foldchange) value is positive and native mean that the gene is up-regulated and down-regulated in the CK sample, respectively. (XLSX 23 kb)

Additional file 2: **Table S2.** The interaction information of rice root differential genes and co-expressed lncRNAs in cis. (XLSX 15 kb)

Additional file 3: **Table S6.** Pathways and proportions after KEGG (Kyoto Encyclopedia of Genes and Genomes) analysis of differentially expressed target genes in cis in the root. KEGG pathways significantly enriched in our experiments were displayed. (XLS 14 kb)

Additional file 4: **Table S3.** The interaction information of rice root differential genes and co-expressed lncRNAs in trans. (XLSX 870 kb)

Additional file 5: **Table S7.** Pathways and proportions after KEGG (Kyoto Encyclopedia of Genes and Genomes) analysis of differentially expressed target genes in trans in the root. KEGG pathways significantly enriched in our experiments were displayed. (XLS 14 kb)

Additional file 6: **Figure S1.** Scatter plot of KEGG pathway enrichment statistics. Rich Factor is the ratio of differentially expressed gene numbers annotated in this pathway term to all gene numbers annotated in this pathway term. Greater Rich Factor means greater intensiveness. *q*-value is corrected *p*-value ranging from 0~ 1, and its less value means greater intensiveness. We just display the top 20 pathway terms enriched by KEGG database. (TIFF 646 kb)

Additional file 7: **Table S4.** The qPCR used transcripts and their primers. We made a random selection of 10 genes in the genes’ data of lncRNA-seq. According to the random criteria, so we have selected both the significantly different expressing part and the nonsignificantly part to enhance the reliability of the results by lncRNA-seq. (XLS 15 kb)

Additional file 8: **Figure S2.** Comparison of the log_2_ (FC) of 10 selected transcripts using RNA-Seq and qRT-PCR. (TIFF 15 kb)

Additional file 9: **Table S5.** The differentially expressed mRNAs by lncRNA-seq. (XLS 831 kb)

## Abbreviations

Cd: Cadmium
CK_R: The rice root in normal condition
FPKMs: Fragments per kb for a million reads
GO: Gene Ontology
KEGG: Kyoto Encyclopedia of Genes and Genomes
lncRNAs: Long non-coding RNAs
SY_R: The rice root in Cd stress

## References

[1] Bazin J, Baileyserres J (2015). Emerging roles of long non-coding RNA in root developmental plasticity and regulation of phosphate homeostasis. Front Plant Sci.

[2] Liu J, Wang H, Chua NH (2015). Long non coding RNA transcriptome of plants. Plant Biotechnol J.

[3] Cabili MN, Trapnell C, Goff L (2011). Integrative annotation of human large intergenic noncoding RNAs reveals global properties and specific subclasses. Genes Dev.

[4] Li T, Wang S, Wu R, Zhou X (2012). Identification of long nonprotein coding RNAs in chicken skeletal muscle using next generation sequencing. Genomics.

[5] Zhou ZY, Li AM, Adeola AC (2014). Genome-wide identification of long intergenic noncoding RNA genes and their potential association with domestication in pigs. Genome Biology and Evolution.

[6] Derrien T, Johnson R, Bussotti G (2012). The GENCODE v7 catalog of human long noncoding RNAs: analysis of their gene structure, evolution, and expression. Genome Res.

[7] Nam JW, Bartel DP (2012). Long noncoding RNAs in C. Elegans. Genome Res.

[8] Pauli A, Valen E, Lin MF (2012). Systematic identification of long noncoding RNAs expressed during zebrafish embryogenesis. Genome Res.

[9] Lv Y, Liang Z, Min G (2016). Genome-wide identification and functional prediction of nitrogen-responsive intergenic and intronic long non-coding RNAs in maize (Zea mays L.). BMC Genomics.

[10] Heo JB, Sung S (2011). Vernalization-mediated epigenetic silencing by a long intronic noncoding RNA. Science.

[11] Bardou F, Ariel F, Simpson C (2014). Long noncoding RNA modulates alternative splicing regulators in Arabidopsis. Dev Cell.

[12] Garima B, Neetu G, Shailesh S, et al. Present scenario of long non-coding RNAs in plants. Non-coding RNA. 2017; 10.3390/ncrna3020016. 10.3390/ncrna3020016 PMC5831932 29657289

[13] Liu X, Hao L, Li D (2015). Long non-coding RNAs and their biological roles in plants. GPB.

[14] Swiezewski S, Liu F, Magusin A, Dean C (2009). Cold-induced silencing by long antisense transcripts of an Arabidopsis polycomb target. Nature.

[15] Wang H, Chung PJ, Liu J (2014). Genome-wide identification of long non coding natural antisense transcripts and their responses to light in Arabidopsis. Genome Res.

[16] Li L, Eichten SR, Shimizu R (2014). Genome-wide discovery and characterization of maize long non-coding RNAs. Genome Biol.

[17] Xin M, Wang Y, Yao Y (2011). Identification and characterization of wheat long non-protein codingRNAs responsive to powdery mildew infection and heat stress by usingmicroarray analysis and SBS sequencing. BMC Plant Biol.

[18] Shuai P, Liang D, Tang S (2014). Genome-wide identification and functional prediction of novel and drought-responsive lincRNAs in Populus trichocarpa. J Exp Bot.

[19] Hao Z, Fan C, Cheng T, Su Y, Wei Q, Li G (2015). Genome-wide identification, characterization and evolutionary analysis of long intergenic noncoding RNAs in cucumber. PLoS One.

[20] Wang J, Yu W, Yang Y (2015). Genome-wide analysis of tomato long non-coding RNAs and identification as endogenous target mimic for microRNA in response to TYLCV infection. Sci Rep.

[21] Zou C, Wang Q, Lu C (2016). Transcriptome analysis reveals long noncoding RNAs involved in fiber development in cotton (Gossypium arboreum). Sci China Life Sci.

[22] Xin M, Wang Y, Yao Y (2011). Identification and characterization of wheat long non-protein coding RNAs responsive to powdery mildew infection and heat stress by using microarray analysis and SBS sequencing. BMC Plant Biol.

[23] Chen J, Quan M, Zhang D (2015). Genome-wide identification of novel long non-coding RNAs in Populus tomentosa tension wood, opposite wood and normal wood xylemby RNA-seq. Planta.

[24] Wen J, Parker BJ, Weiller GF (2007). In silico identification and characterization of mRNA like noncoding transcripts in Medicago truncatula. Silico Biol.

[25] Zhang YC, Liao JY, Li ZY (2014). Genome-wide screening and functional analysis identify a large number of long noncoding RNAs involved in the sexual reproduction of rice. Genome Biol.

[26] Liu TT, Zhu D, Chen W (2013). A global identification and analysis of small nucleolar RNAs and possible intermediate-sized non-coding RNAs in Oryza sativa. Mol Plant.

[27] Wang M, Chen W, Peng C (2015). Risk assessment of cd polluted paddy soils in the industrial and town ship areas in Hunan. Southern China Chemosphere.

[28] Fei H, Liu Q, Li Z (2015). RNA-Seq analysis of Rice roots reveals the involvement of post-transcriptional regulation in response to cadmium stress. Front Plant Sci.

[29] Jin J, Liu J, Wang H (2013). PLncDB: plant long non-coding RNA database. Bioinformatics.

[30] Zhu QH, Stephen S, Taylor J (2014). Long noncoding RNAs responsive to fusarium oxysporum infection in Arabidopsis thaliana. New Phytol.

[31] Amor BB, Wirth S, Merchan F (2009). Novel long non-protein coding RNAs involved in Arabidopsis differentiation and stress responses. Genome Res.

[32] Song D, Yang Y, Yu B (2009). Computational prediction of novel non-coding RNAs in Arabidopsis thaliana. BMC Bioinf.

[33] Zhu B, Yang Y, Li R (2015). RNA sequencing and functional analysis implicate the regulatory role of long non-coding RNAs in tomato fruit ripening. J Exp Bot.

[34] Boerner S, McGinnis KM (2012). Computational identification and functional predictions of long noncoding RNA in Zea mays. PLoS One.

[35] Ren H, Wang G, Chen L (2016). Genome-wide analysis of long non-coding RNAs at early stage of skin pigmentation in goats (Capra hircus). BMC Genomics.

[36] The Nipponbare reference Genome ftp://ftp.ensemblgenomes.org/pub/release-21/plants/fasta/oryza_sativa/dna. Accessed 18 Dec. 2013.

[37] Trapnell C, Pachter L, Salzberg SL (2009). TopHat: discovering splice junctions with RNA-Seq. Bioinformatics.

[38] Guttman M, Garber M, Levin JZ, Donaghey J, Robinson J, Adiconis X, Fan L, Koziol MJ, Gnirke A, Nusbaum C, Rinn JL, Lander ES, Regev A (2010). Ab initio reconstruction of celltype-specific transcriptomes in mouse reveals the conserved multi-exonic structure of lincRNAs. Nat Biotechnol.

[39] Trapnell C, Williams BA, Pertea G, Mortazavi A, Kwan G, van Baren MJ, Salzberg SL, Wold BJ, Pachter L (2010). Transcript assembly and quantification by RNA-Seq reveals unannotated transcripts and isoform switching during cell differentiation. Nat Biotechnol.

[40] Kong L, Zhang Y, Ye ZQ, Liu XQ, Zhao SQ, Wei L, Gao G (2007). CPC: assess the protein-coding potential of transcripts using sequence features and support vector machine. Nucleic Acids Res.

[41] Mistry J, Bateman A, Finn RD (2007). Predicting active site residue annotations in the Pfam database. BMC Bioinformatics.

[42] Trapnell C, Roberts A, Goff L, Pertea G, Kim D, Kelley DR, Pimentel H, Salzberg SL, Rinn JL, Pachter L (2012). Differential gene and transcript expression analysis of RNA-seq experiments with TopHat and cufflinks. Nat Protoc.

[43] Langmead B, Trapnell C, Pop M, Salzberg SL (2009). Ultrafast and memory-efficient alignment of short DNA sequences to the human genome. Genome Biol.

[44] Young MD, Wakefield MJ, Smyth GK, Oshlack A (2010). Gene ontology analysis for RNA-seq: accounting for selection bias. Genome Biol.

[45] Mao X, Cai T, Olyarchuk JG, Wei L (2005). Automated genome annotation and pathway identification using the KEGG Orthology (KO) as a controlled vocabulary. Bioinformatics.

[46] Feng J, Bi C, Clark BS, Mady R, Shah P, Kohtz JD (2006). The Evf-2 noncoding RNAis transcribed from the Dlx-5/6 ultraconserved region and functions as a Dlx-2 transcriptional coactivator. Genes Dev.

[47] Rinn JL, Kertesz M, Wang JK, Squazzo SL, Xu X, Brugmann SA, Goodnough LH, Helms JA, Farnham PJ, Segal E, Chang HY (2007). Functional demarcation of active and silent chromatin domains in human HOX loci by noncoding RNAs. Cell.

[48] Thakur N, Tiwari VK, Thomassin H, Pandey RR, Kanduri M, Gondor A, Grange T, Ohlsson R, Kanduri C (2004). An antisense RNA regulates the bidirectional silencing property of the Kcnq1 imprinting control region. Mol Cell Biol.

[49] Sleutels F, Zwart R, Barlow DP (2002). The non-coding air RNA is required for silencing autosomal imprinted genes. Nature.

[50] Marquardt S, Raitskin O, Wu Z, Liu F, Sun Q, Dean C (2014). Functional consequences of splicing of the antisense transcript COOLAIR on FLC transcription. Mol Cell.

[51] Shahryari A, Jazi MS, Samaei NM, Mowla SJ (2015). Long non-coding RNA SOX2OT. Expression signature, splicing patterns, and emerging roles in pluripotency and tumorigenesis. Front Genet.

[52] Chen FC, Pan CL, Lin HY (2014). Functional implications of RNA splicing for human long intergenic noncoding RNAs. Evol Bioin form Online.

[53] Tang M, Mao D, Xu L, Li D, Song S, Chen C (2014). Integrated analysis of miRNA and mRNA expression profiles in response to cd exposure in rice seedlings. BMC Genomics.

[54] DalCorso G, Farinati S, Maistri S, Furini A (2008). How plants cope with cadmium:staking all on metabolism and gene expression. J Integr Plant Biol.

[55] Fernández R, Bertrand A, Reis R, Mourato MP, Martins LL, González A (2013). Growth and physiological responses to cadmium stress of two populations of Dittrichia viscosa (L.) Greuter. J Hazard Mater.

[56] Jiang H-p, Gao B-b, Li W-h (2013). Physiological and biochemical responses of Ulva prolifera and Ulva linza to cadmium stress. Sci World J.

[57] Lu S, Li L (2008). Carotenoid metabolism: biosynthesis, regulation, and beyond. J Integr Plant Biol.

[58] Li S-W, Leng Y, Feng L, Zeng X-Y (2014). Involvement of abscisic acid in regulating antioxidative defense systems and IAA-oxidase activity and improving adventitious rooting in mung bean [Vigna radiata (L.) Wilczek] seedlings under cadmium stress. Environ Sci Pollut Res.

[59] Hsu YT, Kao CH (2003). Role of abscisic acid in cadmium tolerance of rice (Oryza sativa L.) seedlings. Plant Cell Environ.

[60] Rodriguez-Serrano M, Romero-Puertas MC, Pazmino DM, Testillano PS, Risueno MC, Rio LA (2009). Cellular response of pea plants to cadmium toxicity: crosstalk between reactive oxygen species, nitric oxide, and calcium. Plant Physiol.

[61] Zhai R, Feng Y, Wang H (2013). Transcriptome analysis of rice root heterosis by RNA-Seq. BMC Genomics.

