# Supplementary figures and images for: Genome-wide analysis of long non-coding RNAs affecting roots development at an early stage in the rice response to cadmium stress
Source: BMC Genomics. 2018 Jun 15;19:460. doi: 10.1186/s12864-018-4807-6 (PMC6002989; doi:10.1186/s12864-018-4807-6)

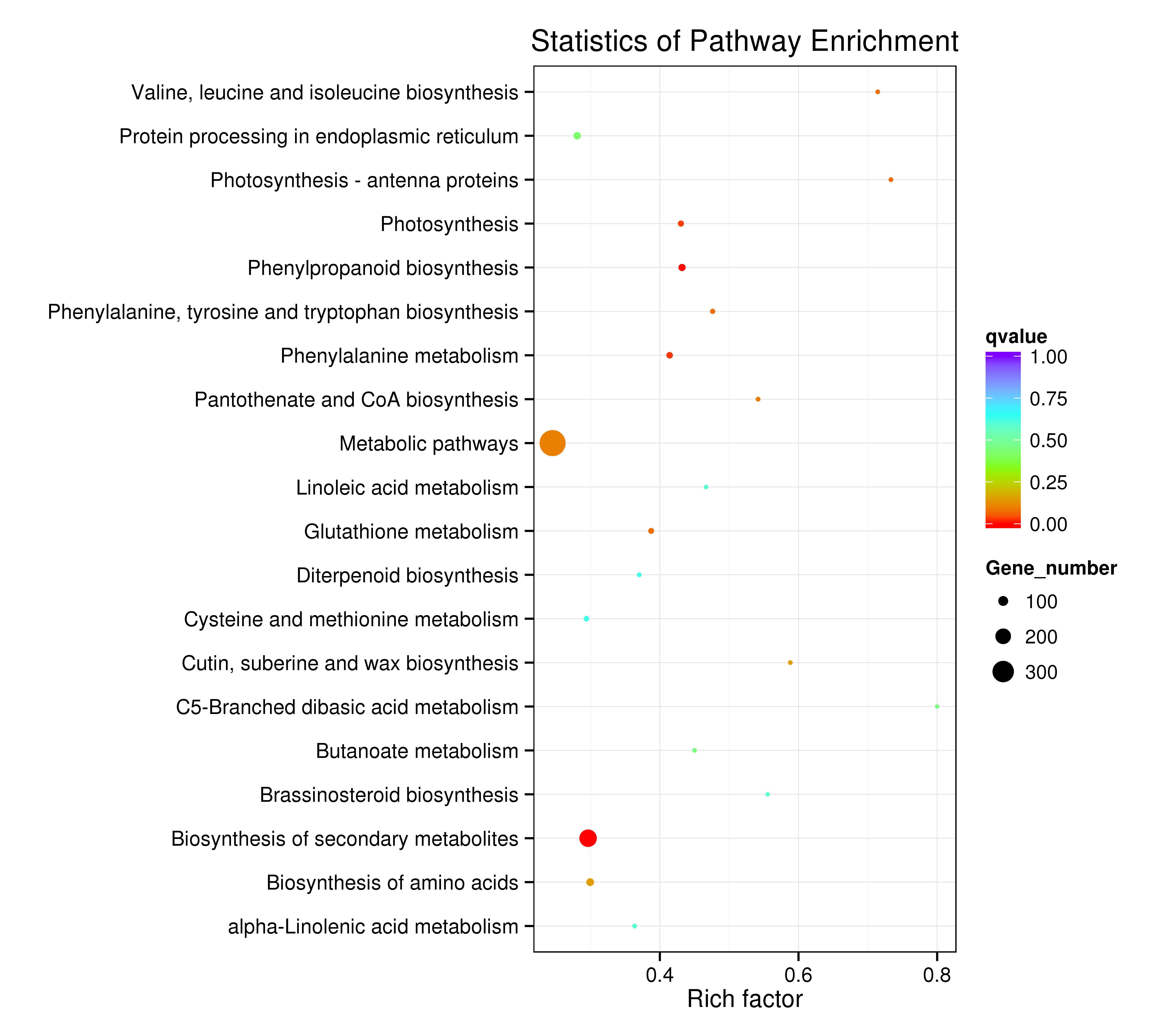

Supplement: Supplementary file 6 — Figure S1. Scatter plot of KEGG pathway enrichment statistics. Rich Factor is the ratio of differentially expressed gene numbers annotated in this pathway term to all gene numbers annotated in this pathway term. Greater Rich Factor means greater intensiveness. q-value is corrected p-value ranging from 0~ 1, and its less value means greater intensiveness. We just display the top 20 pathway terms enriched by KEGG database. (TIFF 646 kb) [file 12864_2018_4807_MOESM6_ESM.tiff]

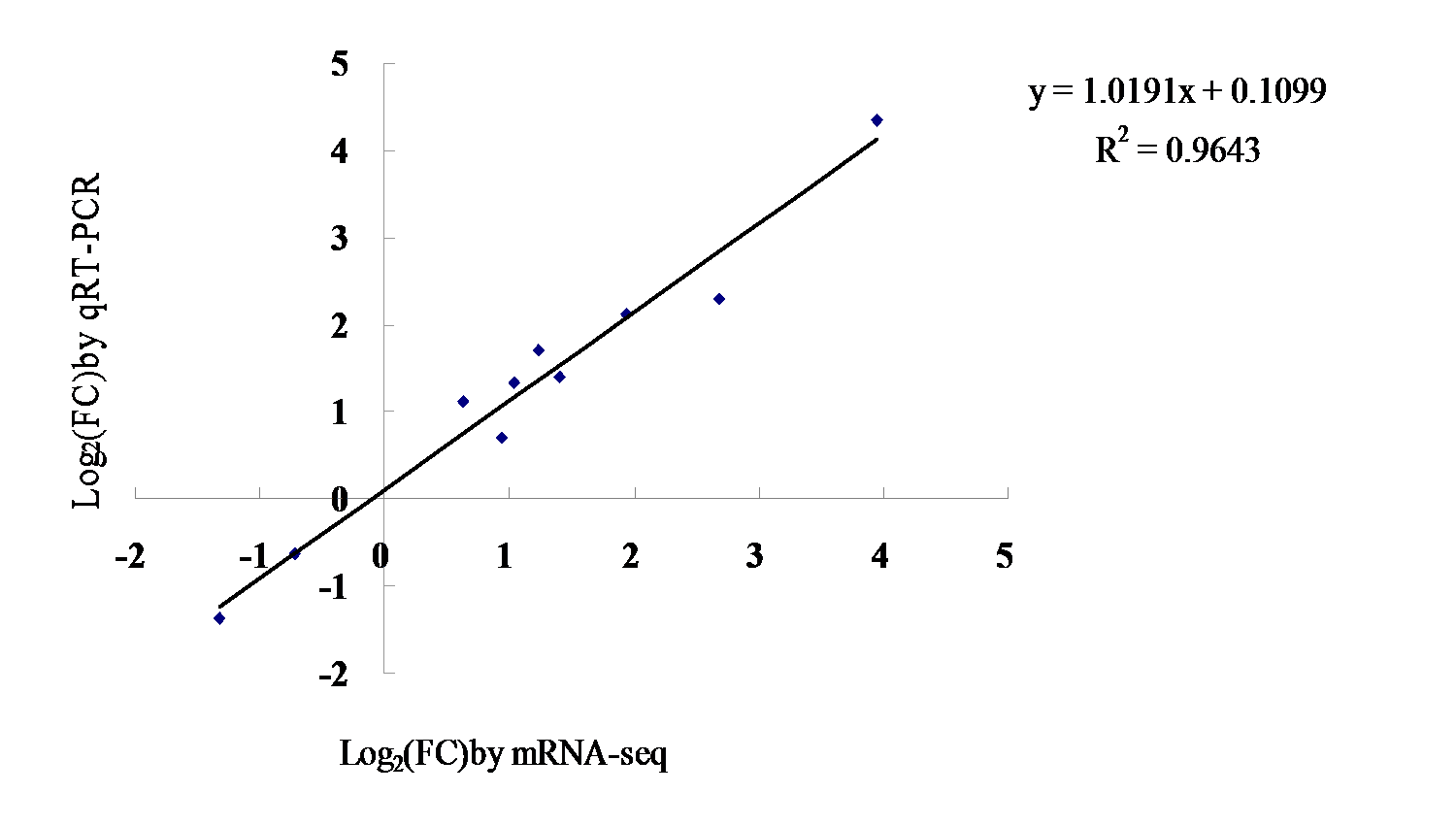

Supplement: Supplementary file 8 — Figure S2. Comparison of the log2 (FC) of 10 selected transcripts using RNA-Seq and qRT-PCR. (TIFF 15 kb) [file 12864_2018_4807_MOESM8_ESM.tiff]
